# Graphene Oxide Nanosheets Tailored With Aromatic Dipeptide Nanoassemblies for a Tuneable Interaction With Cell Membranes

**DOI:** 10.3389/fbioe.2020.00427

**Published:** 2020-05-08

**Authors:** Giuseppe Trapani, Viviana Carmela Linda Caruso, Lorena Maria Cucci, Francesco Attanasio, Giovanni Tabbì, Giuseppe Forte, Diego La Mendola, Cristina Satriano

**Affiliations:** ^1^Scuola Superiore di Catania, University of Catania, Catania, Italy; ^2^Department of Chemical Sciences, Nano Hybrid BioInterfaces Lab (NHBIL), University of Catania, Catania, Italy; ^3^Institute of Crystallography - National Council of Research, Catania, Italy; ^4^Department of Pharmaceutical Sciences, University of Catania, Catania, Italy; ^5^Department of Pharmacy, University of Pisa, Pisa, Italy

**Keywords:** self-assembly, supported lipid bilayers, copper, diphenylalanine, dityrosine, peptide nanotubes, AFM, QCM-D.

## Abstract

Engineered graphene-based derivatives are attractive and promising candidates for nanomedicine applications because of their versatility as 2D nanomaterials. However, the safe application of these materials needs to solve the still unanswered issue of graphene nanotoxicity. In this work, we investigated the self-assembly of dityrosine peptides driven by graphene oxide (GO) and/or copper ions in the comparison with the more hydrophobic diphenylalanine dipeptide. To scrutinize the peptide aggregation process, in the absence or presence of GO and/or Cu^2+^, we used atomic force microscopy, circular dichroism, UV–visible, fluorescence and electron paramagnetic resonance spectroscopies. The perturbative effect by the hybrid nanomaterials made of peptide-decorated GO nanosheets on model cell membranes of supported lipid bilayers was investigated. In particular, quartz crystal microbalance with dissipation monitoring and fluorescence recovery after photobleaching techniques were used to track the changes in the viscoelastic properties and fluidity of the cell membrane, respectively. Also, cellular experiments with two model tumour cell lines at a short time of incubation, evidenced the high potential of this approach to set up versatile nanoplatforms for nanomedicine and theranostic applications.

## Introduction

In the past decade, the use of engineered carbon-based nanomaterials for technological applications has attracted much interest in the field of nanomedicine. In particular, several salient features of graphene make it a potential candidate for biological and biomedical applications (Dasari Shareena et al., 2018). For instance, graphene has been reported to have antibacterial, antiplatelet, and anticancer activities (Gurunathan and Kim, 2016). However, in any kind of applications related to human welfare, the biocompatibility and the toxicity of graphene are fundamental issues that require thorough investigation.

Graphene oxide (GO) has long been considered hydrophilic, owing to its excellent water dispersity (differently than hydrophobic graphene), with the nanosheets separated from each other due to the electrostatic repulsion between the negative charges present on the GO surface (Mathesh et al., 2013). Hybrid metal-GO materials have been developed by binding Cu^2+^ with the negatively charged groups on GO (Mathesh et al., 2013). The toxic effects of graphene can be influenced by physicochemical properties such as size and distribution, surface charge, surface area, layer number, lateral dimensions, surface chemistry, purity, surface functional groups, and shape (Mao et al., 2013). The mechanisms by which GO and reduced GO show toxicity against cells and bacteria include the membrane stress caused by direct contact with sharp nanosheets, ensuing enhanced production of reactive oxygen species (ROS), the loss of plasma membrane structural integrity associated with a strong physical interaction of GO with the phospholipid bilayer (Lammel et al., 2013), apoptosis and autophagy (Jia et al., 2019). Noteworthy, graphene-based materials tailored at their surface with biomolecules may considerably change their response at the biointerface (Zhang et al., 2013; He et al., 2017). In general, the surface “decoration” of nanomaterials by peptide molecules is a useful approach to increase their biocompatibility by a biomimetic approach as well as to enhance their biological effects (Forte et al., 2014; Naletova et al., 2019).

Peptide-based self-assembly offers new routes to fabricate various nanostructures including nanotubes, nanowires, nanospheres, nanofibrils, vesicles and hydrogels (Adler-Abramovich and Gazit, 2014), which are of interest in materials science, energetics and nanomedicine applications (Gan and Xu, 2017; Schnaider et al., 2017; Tesauro et al., 2019). Aromatic amino acids, such as phenylalanine (Phe, F) and tyrosine (Tyr, Y), are known to assemble into helical nanotube or nanofiber structures, through a process mostly driven by π–π stacking, charge transfer and H-bonding (Reches and Gazit, 2003; Menard-Moyon et al., 2015). This ordered structuring can be influenced by divalent cations, especially copper (Jin et al., 2011), and 2D graphene-based nanostructures (Supplementary Figure 1). Both physical and chemical adsorption were found prominent in the interaction of Phe with graphene oxide (GO), while Tyr exhibited a strong chemisorption (Mallineni et al., 2016).

In the last few years, nanostructures based on the diphenyl peptide (FF) (Gorbitz, 2006), the core recognition motif of the Alzheimer’s β-amyloid polypeptide, have been deeply investigated to get mechanistic insight into the self-assembly of amyloid fibrils (Wang et al., 2016). These studies paved the way for new technological applications of dipeptide-based nanostructures, having high stiffness and stability in extreme chemical and physical conditions (Kol et al., 2005).

Unlike the most popular analogous of FF, there are only few reports on ordered assemblies of the dityrosine peptide (YY) (Jang et al., 2014; Roviello et al., 2018) and, to the best of our knowledge, to date, there is no report regarding GO functionalized with aromatic peptide-based supramolecules. Moreover, the hybrid biointerface between dipeptide nanostructures and cell membranes has been only marginally addressed so far (Marchesan et al., 2015; Schnaider et al., 2017).

We demonstrate here that the different surface decoration of the graphene-based 2D nanosheets by the two dipeptides allows for a modulated interaction with artificial cell membranes made of 1-palmitoyl-2-oleoyl-sn-glycero-3-phosphocholine (POPC) supported lipid bilayers (SLBs). Specifically, in this work we scrutinized the self-assembly of FF and YY dipeptides in the absence and/or presence of GO nanosheets as well as of copper ions (Cu^2+^). The morphology of the peptide assemblies was scrutinized by atomic force microscopy (AFM), while the aggregation process of the peptides in the presence of copper and/or GO was studied by circular dichroism (CD), UV–visible and fluorescence spectroscopies, to assess the conformational features, the copper complex formation and the energy transfer processes in the aggregates, respectively. Moreover, the coordination environment of the peptides-copper complexes grown in the presence of GO was investigated by means of electron paramagnetic resonance (EPR) spectroscopy. The perturbative effect by the hybrid nanomaterials on SLBs model cell membranes was studied by means of quartz crystal microbalance with dissipation monitoring (QCM-D) and fluorescence recovery after photobleaching (FRAP) techniques, to analyze, upon the formation of the hybrid SLB-dipeptide biointerface, the viscoelastic as well as the fluidity features of the artificial cell membrane, respectively.

The obtained results pointed out the different surface decoration of the graphene nanosheets, with the peptide molecules that selectively gather at their edges (FF) or basal planes (YY), with significant changes observed for the nanoassemblies grown in the presence of copper ions. Proof-of-work cellular experiments on human tumor (neuroblastoma and prostatic) cells highlighted the promising potential of these platforms for theranostic applications.

## Materials and Methods

### Chemicals

Phe-Phe (FF) and Tyr-Tyr (YY) dipeptides were obtained from CASLO ApS (Denmark). 1,1,1,3,3,3-hexafluoro-2-propanol (HFIP), copper sulfate and Thioflavin-T (Th-T) were purchased from Sigma Aldrich (Italy). Ultrapure Millipore water (18.2 mΩ cm at 25°C) was used to prepare all aqueous solutions. GO was purchased from Graphenea Inc., (United States). Small unilamellar vesicles (SUVs) were prepared from 1-palmitoyl-2-oleoyl-sn-glycero-3-phosphocholine (POPC) and rhodamine-labeled 1,2-dihexadecanoyl-snglycero-3-(Rhod-DHPE) purchased from Avanti Polar Lipids (Alabaster, AL, United States). Phosphate buffer saline (PBS) solution (0.01 M phosphate buffer containing 0.003 M KCl and 0.14 M NaCl, pH 7.4) was prepared from tablets (Sigma). Dulbecco’s modified eagle medium (DMEM)-F12, RPMI 16-40 medium, fetal bovine serum (FBS), streptomycin and L-glutamine, dimethyl sulfoxide (DMSO), bovine serum albumin (BSA), 3-(4,5-dimethylthiazol-2-yl)-2,5-diphenyltetrazolium bromide (MTT) were purchased from Sigma Aldrich.

### Assembling and Physicochemical Characterization of Dipeptide-Based Nanotubes

#### Assembling of Phe-Phe and Tyr-Tyr Hybrids With GO Nanosheets

As received GO was diluted in ultrapure Millipore water to a final concentration of 0.4 mg/mL. The GO sheets with different lateral sizes were prepared by sonication for 120 min using a titanium cup-horn sonicator (Hielscher UP200Ht) at 200 W and 24 KHz. The sonicated GO was centrifuged (13000 rpm, 20 min), to separate nanosheets (sub-micron lateral size) from bulky large sheets (up to several mm of lateral size), most likely assembled in the pellet. The collected supernatant typically contained approximately 0.3 mg/mL of GO, as determined by UV–visible spectroscopy (GO extinction coefficient, ε_230__nm_, of 57.9 mg^–1^ mL cm^–1^). Fresh stock solutions of Phe-Phe and Tyr-Tyr were prepared by dissolving lyophilized form of the peptides in HFIP at a concentration of 100 mg/mL. The peptides stock solutions were diluted to a final concentration of 10^–3^ M in Millipore water, CuSO_4_ (10^–3^ M), GO dispersion (0.34 mg/mL) and GO/CuSO_4_ (0.34 mg/mL, 10^–3^ M). The pH of the solutions was adjusted to 7.4. The solutions were stored at room temperature.

#### Atomic Force Microscopy

Ten microliters of the 10^–3^ M solutions were put on freshly cleaved muscovite mica (Ted Pella, Inc.) and incubated at room temperature for 5 min. After that, the mica surface was washed with 1 mL of Millipore water, dried under a gentle nitrogen stream, and imaged. Scans were recorded using a Cypher AFM instrument (Asylum Research, Oxford Instruments, Santa Barbara, CA, United States), operating in tapping-mode and furnished with a scanner at an XY scan range of 30/40 μm (closed/open loop). Tetrahedral tips, made of silicon and mounted on rectangular 30 μm long cantilevers, were purchased from Olympus (AT240T S, Oxford Instruments). The probes had nominal spring constants of 2 N/m and driving frequencies of 70 kHz. Images with an area of 2 × 2 μm and 5 × 5 μm were scanned, and the sizes of particles were measured using a free tool in the MFP-3DTM offline section analysis software. AFM images were acquired at different aging time, namely 18, 42, 66, 120, and 240 h.

#### Circular Dichroism (CD), UV–Visible and Fluorescence Spectroscopies

CD spectra were obtained at 25°C under a constant flow of nitrogen on a Jasco model 810 spectropolarimeter, calibrated with an aqueous solution of (1R)-(-)-10-camphorsulfonic acid. Measurements were carried using 1 cm path length quartz cuvettes. The CD spectra were obtained in the 190–350 nm wavelength region. CD spectra were acquired at different aging time, namely 18, 42, 66, 120, and 240 h. UV–visible spectroscopy was performed in quartz cuvettes with 1 cm optical path length on a Jasco spectrometer. All the solutions were freshly diluted using Millipore water. Fluorescence studies of all samples were carried out by a Horiba Jobin Yvon Fluoromax spectrometer with a path length of 1 cm. Emission spectra of the samples were recorded at the range of 235–425 nm with an excitation wavelength of 230 nm. All the solutions were freshly diluted using Millipore water. The used concentrations were of 5⋅10^–5^ M for dipeptide, 5⋅10^–5^ M for Cu^2+^ and 17 μg/mL for GO, respectively.

#### Electron Paramagnetic Resonance

EPR measurements were carried out by using a Bruker Elexsys E500 CW-EPR spectrometer driven by a PC running the XEpr program on Linux and equipped with a Super-X microwave bridge, operating at 9.3–9.5 GHz, and a SHQE cavity were used throughout this work. All the EPR spectra of frozen solutions of Cu^2+^ complexes were recorded at 150 K by means of a ER4131VT variable temperature apparatus. EPR magnetic parameters were obtained directly from the experimental EPR spectra, calculating them from the 2nd and 3rd line to avoid second order effects. The instrument settings for the EPR spectra recordings of the copper^2+^-peptide complexes were as follows: number of scans 1–5; microwave frequency 9.344–9.376 GHz; modulation frequency 100 kHz; modulation amplitude 0.2–0.6 mT; time constant 164–327 ms; sweep time 2.8 min; microwave power 20–40 mW; receiver gain 50–60 dB. Copper^2+^-peptide complexes were prepared by adding the appropriate amount of isotopically pure copper, taken from a 0.05 M ^63^Cu(NO_3_)_2_ solution, to the peptide solution. The copper^2+^-peptide complex solutions were prepared at 1:1 metal-to-ligand ratio (10^–3^ M concentration), with the GO concentration fixed to 0.34 mg/mL.

#### Thioflavin (Th-T) Fluorescence Assay for Fibril Formation

A VarioSkan Flash fluorescence 96-well plate reader (Thermo Scientific) was used for the Th-T measurements. To minimize evaporation effects, the wells were sealed with a transparent heat-resistant plastic film. Readings were taken every 10 min, after gentle shaking for 10 s, during a total acquisition time of 2 days. The fluorescence excitation wavelength was set at 440 nm, and emission was detected at 480 nm. All Th-T experiments were carried out at pH 7.4, 37°C and in 10 mM phosphate buffer. The Th-T concentration was 60 μM. The Th-T curves represent the average of three independent experiments, which were each run in quadruplicate.

### Theoretical Calculations

All the calculations were performed in the framework of the DFT theory, using the PBE0 functional (Adamo and Barone, 1999) and the extended 6-311+^∗∗^ basis set (Krishnan et al., 1980; McLean and Chandler, 1980; Binning and Curtiss, 1990). In the simulations FF and YY were considered in the zwitterionic form while GO surface was decorated at the edges with carboxylic acids and modeled on the basis of the analysis performed by Klinowski et al. with hydroxyl and epoxy groups mainly present and randomly distributed on both sides of the sheet (Lerf et al., 1998). Solvent effects were included by means of the Polarizable Continuum Model (PCM) (Klamt and Schüürmann, 1993; Cossi et al., 2003) and optimization with tight optimization criteria was adopted to describe properly the systems.

### Interaction With SLBs and Lipid Lateral Diffusion Measurements

#### Small Unilamellar Vesicles Synthesis

A 5 mg/mL solution of POPC in chloroform, mixed with Rhod-DHPE (1 wt.%), was added in a round-bottom flask and the chloroform was evaporated under a flow of N_2_ while rotating the flask, in order to create a uniform film on the glass wall. The dried lipid film was emulsified by using 10 mM PBS, at room temperature, and vortexed to obtain the vesicles. Afterward, in order to obtain the SUVs dispersion, about 80–100 nm of diameter (Satriano et al., 2010), the dispersion was extruded 13 times through a 100 nm-pore size polycarbonate membrane, followed by another 13 times through a 30 nm-pore size membrane (Avanti Polar Lipids). The stock solution of rhodamine-labeled POPC was stored under N_2_ and used within 2 weeks, according to an established protocol (Satriano et al., 2010).

#### Quartz Crystal Microbalance With Dissipation Monitoring and Supported Lipid Bilayers Formation

QCM-D measurements were carried out in flow mode (100 μL/min) on a Q-sense D 300 setup (Q-Sense AB, Gothenburg, Sweden). Prior to each measurement series, the crystals (SiO_2_-coated AT-cut quartz crystals with a fundamental resonance frequency of 5 MHz) were cleaned by immersion in 10 mM sodium dodecyl sulfate (SDS, >1 h), followed by rinsing with water, drying with nitrogen, and UV–O_3_ treatment (30 min). Frequency shifts were normalized by the overtone number. After stabilization of the baseline, 0.5 mL of a temperature-stabilized vesicle solution (100 μg/mL) was injected in the measurement chamber for the formation of the lipid bilayer on the sensor surface, by the spontaneous rupture/fusion processes following the adsorption of the vesicles onto the hydrophilic silica surfaces of the QCM-D sensor (Satriano et al., 2010, 2012).

#### Fluorescence Recovery After Photobleaching

To carry out the FRAP experiments, glass bottom dishes (Willco Wells) were cleaned by two repeated cycles of 20 min UV–O_3_ treatment, with water rinsing and N_2_ flow drying in between and at the end of the treatment. Subsequently, the SUVs (100 μg/mL) were allowed to adsorb onto the glass surface of the dishes for 30 min, to form fluid SLBs by the spontaneous rupture/fusion processes following the adsorption of the vesicles onto the hydrophilic glass surfaces.(Satriano et al., 2010, 2012) After the rinsing with buffer solution, the SLBs were incubated with the Phe-Phe and Tyr-Tyr samples. After the treatment time of 15 min, the samples were washed with buffer and transferred to the sample holder stage of the confocal microscope. Time-solved snapshots at an interval of 10 s were acquired in the following sequence: three images before bleach, bleaching at the maximum Ar laser power (98%), other ten micrographs after the bleaching. By translating the sample stage, five spots per substrate were photobleached in a given experiment. Fluorescence recovery curves were analyzed by ImageJ software (FRAP Profiler macro), and the data were normalized to the initial pre-photobleach value. For each sample, the emission recorded from the bleached spots was compared with that obtained from contiguous non-bleached areas. For the calculation of the lipid diffusion coefficient (D), the Axelrod and Soumpasis equations (Axelrod et al., 1976; Soumpasis, 1983) that relate to the half time of recovery (t_1/2_) and to the nominal radius of the user-defined bleach spot (r) for a pure isotropic diffusion model were used: D = (0.224 r^2^)/t_1/2_.

### Interaction With Cells and Cytotoxicity Studies

#### Cell Culture

SH-SY5Y neuroblastoma cells were cultured in Dulbecco’s modified Eagle medium (DMEM-F12) supplemented with 10% FBS and 2 mM glutamine; PC-3 prostate cancer cells were cultured in RPMI 16-40 medium supplemented with 10% FBS. 100 U penicillin and 0.1 mg/mL streptomycin were added in all the used cell media. Cells were grown in tissue-culture treated Corning^®^ flasks (Sigma-Aldrich, St. Louis, MO, United States) under a humidified atmosphere of air/CO_2_ (95:5) at 37°C in an incubator (Heraeus Hera Cell 150C incubator).

#### Cell Viability Assay (MTT)

To perform the cytotoxicity assay, cells were plated in three different 96-well plates in complete medium for 24 h, at a density of 15 × 10^3^ cells per well for SH-SY5Y and 10 × 10^3^ cells per well for PC-3, respectively. Afterword, cells were treated with FF (1⋅10^–4^ M, 5⋅10^–5^ M, and 2⋅10^–5^ M), Cu^2+^ (1⋅10^–4^ M, 5⋅10^–5^ M, and 2⋅10^–5^ M) and GO (28, 14, and 5.6 μg/mL for GO) for 1 h in medium supplemented with 1%. Cytotoxicity was determined at 37°C by using the tetrazolium dye 3-(4,5-dimethylthiazol-2-yl)-2,5-diphenyltetrazolium bromide (MTT). After 1 h of incubation, the enzymatic reduction of MTT to the insoluble purple formazan product was detected by dissolving the crystals with 100 μL of dimethyl sulfoxide and thus measuring the absorbance at 570 nm by Varioscan spectrophotometer. The experiments were performed in triplicate and the results are presented as the means ± SEM.

## Results and Discussion

**FIGURE 1:**
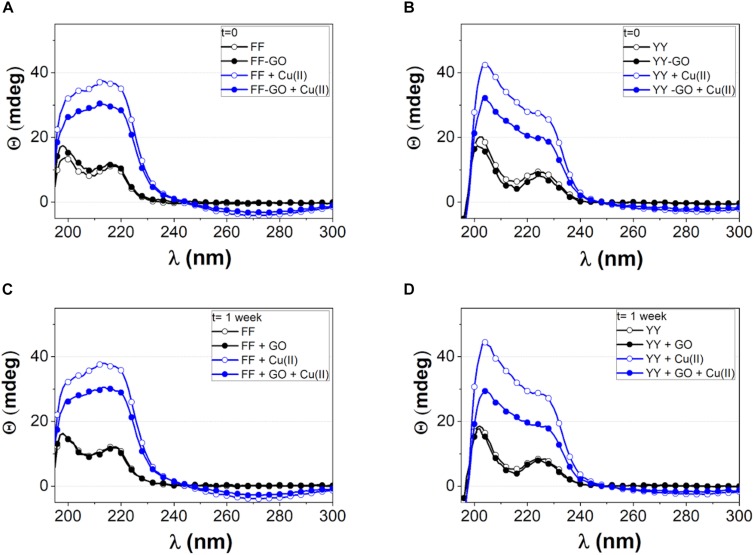
CD spectra of FF **(A, C)** and YY **(B, D)** dipeptides freshly dissolved in water **(A, B)** and after 1 week of aging since dissolution **(C, D)**. Free peptides (open symbols) or GO-driven (solid symbols) aggregation was followed both in the absence (black symbols and lines) and in the presence of CuSO_4_ (blue symbols and lines).

Since the growth process of self-assembling molecules follows kinetics and thermodynamics paths that depend on the used experimental conditions (Zhao et al., 2008), we scrutinized by CD spectroscopy the conformational features of FF and YY dipeptides dissolved in ultrapure water (concentration Figure 1 displays the characteristic bands of beta-sheet secondary structures (Crisma et al., 2009) for the ordered stacking of aromatic rings in FF (Mishra and Chauhan, 2011). Specifically, two strong positive bands are visible, centered at 197 nm (π–π^∗^ transition) and near 220 nm (n–π^∗^ transition), respectively. To note, the minimum at 218 nm beta-sheet conformation is hidden by the aromatic transition centered at 220 nm. In the case of YY (Figure 1B), the two peaks red-shifted to 200 nm and 225 nm.

For both FF and YY, the spectra of the self-assembled aggregates grown in the presence of GO are similar to those of the free dipeptides, with no significant differences found between the freshly dissolved peptides (Figures 1A,B) and the aged solution (Figures 1C,D).

In the presence of divalent copper ions (1:1 molar ratio with the peptides), an intensity enhancement of the CD bands was observed, in parallel with the appearance (especially for FF) of a broad negative band peaked near 280 nm, due to the contribution by aromatic side chains (Gupta et al., 2007). Such an increase of CD signal intensity was more evident in the free dipeptide assemblies than in the peptide-GO hybrids, thus suggesting: (i) a metal-induced extra structuring effect e.g., head-to-tail arrangement of the peptide molecules driven by charge transfer (Jin et al., 2011), (ii) a strong interaction between the dipeptide molecules and the GO nanosheets. Also in the presence of copper ions, the CD spectra carried out after 1 week did not show significant differences with respect to those performed at *t* = 0, suggesting that solution aging did not affect the secondary structure of the aggregates at the used experimental conditions.

In parallel to CD, the UV–visible and fluorescence spectra of the dipeptides were acquired at different times and up to 1 week since the sample dissolution in milliQ water (*t* = 0) to observe spectral features characteristic of the aggregates (Supplementary Figures 2, 3)confirm. For both FF (Supplementary Figure S2, upper panel) and YY (Supplementary Figure S2, lower panel), a widewide absorption band is observed in the 190-230 nm range, due to π → π^∗^ transitions (Antosiewicz and Shugar, 2016; Prasad et al., 2017). The fluorescence spectra of FF in Supplementary Figure S3 display (upper panel) show a peak around 284 nm and another wide centered at 347 due to intramolecular aromatic π–π interactions characteristic of FF nanotubes aggregates (Gan and Xu, 2017). The fluorescence spectra of YY (Supplementary Figure S3, lower panel) show a large peak at 306 nm, characteristic of tyrosine emission, with a shoulder around 340 nm, that may be related to aromatic stacking interaction.

The addition of Cu^2+^ quenches the fluorescence emission of both dipeptides due to the complex formation (Li et al., 2019). No significant changes are displayed in the maximum of absorption and emission bands for both dipeptides; only an increase of intensity indicative of larger aggregates formation. The presence of GO also quenches the emission of both dipeptides, according to literature (Li et al., 2012). In particular, the quenching mechanism between GO and tyrosine is known to be mainly static quenching, combined with dynamic quenching (Förster resonance energy transfer), where an important role is played by electrostatic interaction during quenching (Shi et al., 2018).

Similarly to what detected by CD spectra, the presence of copper during the growth of the peptide aggregates induces the appearance of the aromatic side-chains absorption, in the 230–300 nm range. No significant changes in the aromatic side-chains absorption (signature of differences in stacking interactions and hence in the supramolecular assembly) were observed during the aging time, but only a general increase of absorption for the signal relative to the peptide bond.

Both the enhancement of absorption with aging time as well as the quenching of the intrinsic amino acid fluorescence (Li et al., 2012) pointed to electron charge transfer processes, owing to an actual and strong interaction between the peptide molecules and the GO. The Th-T assay excluded the fibrillary amyloid-like nature of these aggregates. In fact, fluorescence emission spectra of Th-T, which undergo a red shift upon incorporation into β-sheet amyloid structures, remained almost unchanged for the whole period of monitoring, thus indicating that no amyloid-like fibrils formation occurred under experimental conditions used (Supplementary Figure S4).

**FIGURE 2:**
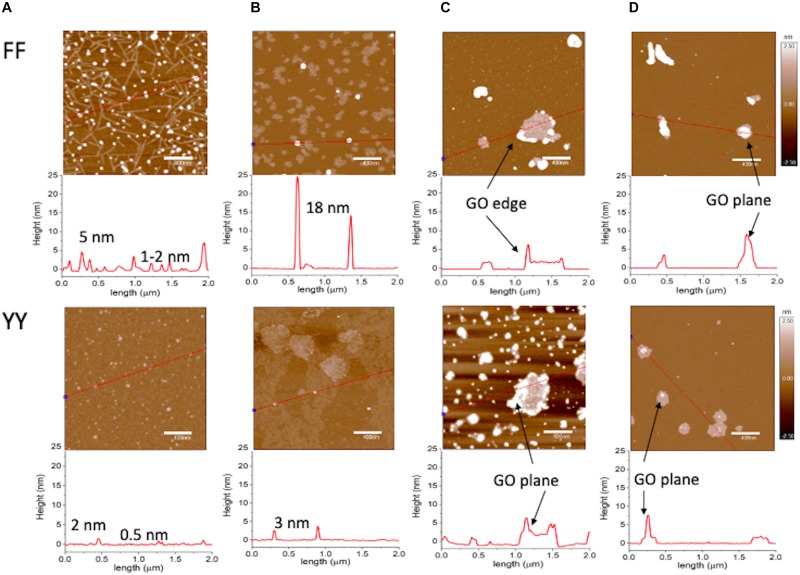
AFM topography images recorded in AC mode in air for dipeptides after 1-week self-assembly in: **(A)** water (10^–3^ *M* peptide concentration); **(B)** CuSO_4_ (10^–3^ *M*); **(C)** GO aqueous dispersion (340 μg/mL); **(D)** GO (340 μg/mL) in CuSO_4_ (10^–3^ *M*). *z*-scale = 5 nm. Below each panel, the section analysis curve corresponding to the red line drawn in the micrographs is reported. Representative images of experiments repeated in triplicate.

AFM analyses confirmed the strong effect of copper ions on the assembly process for FF and YY, as well as the different GO-driven ordered arrangement of the dipeptide nanostructures (Figure 2). The supramolecular assemblies of free dipeptides (Figure 2A) deposited onto the mica substrate displayed, in the case of FF, a bimodal distribution with tubular structures (about 500 nm long and 1–2 nm in height) and round features (up to 5 nm in height), respectively. As to YY, rounded nanoaggregates were predominantly found for the free peptide molecules. The presence of copper (Figure 2B) strongly affected the growth of FF aggregates, with patched domain of supramolecular assemblies, few hundreds of nanometers in lateral size and 1,2 nm in height, instead of the nanotubes. Also, much taller rounded features were formed, with heights up to 20–25 nm. For YY + Cu^2+^, flat and extended regions comparable to those formed by FF + Cu^2+^ were observed. As to the dipeptide molecules self-assembled in the presence of GO (Figure 2C), the hydrophobic FF molecules preferentially gathered at the edges of the nanosheets, which contain a relatively low number of carbonyls, quinones, carboxylic acids, phenols, and lactones (Vacchi et al., 2018). Contrarily, a massive coverage by the biphenol-like YY molecules was found on the basal planes of the nanosheets, where GO is rich in phenol and epoxide groups, and the adsorption of Tyr is expected to be driven by electrostatic interactions (Nassef et al., 2018). Also the dipeptide-GO + Cu^2+^systems (Figure 2D) displayed different topographies than the corresponding features assembled in the absence of the metal ion. Specifically, FF molecules accumulated on the GO planes, likely due to the bridged chelating effect of copper ions that could bind to the hydroxyl groups on the GO planes (Mathesh et al., 2013), while much less YY aggregates, still preferentially localized at the GO basal planes, could be detected.

According to a simple qualitative model, the most preferential interactions between GO and the aromatic dipeptides are hydrophobic forces (π–π stacking and hydrophobic effect) that occur on the graphitic zones of GO (unoxidized region) (Yan et al., 2015). In the oxidized region, there also exist the hydrophobic interactions on sp^2^ clusters, although they may be hindered by surrounding sp^3^ zones, which were more accessible to YY then FF, through H-bonding or electrostatic effects. In the presence of copper, for FF one can invoke a bridged chelating effect of the divalent ions that bind to the hydroxyl groups on the GO planes (Mathesh et al., 2013). As to YY-GO + Cu^2+^, the observed decreased decoration of the GO nanosheets probably is due to a competitive role of copper ions in the electrostatic-driven interaction with the hydrophilic groups on the nanosheets.

**FIGURE 3:**
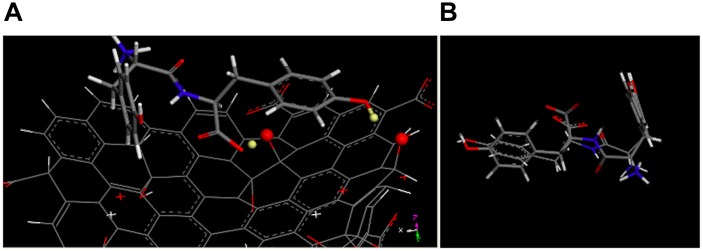
**(A)** Optimized geometries of YY (stick) onto the basal plane of GO (line), with the hydrogen atoms (balls) involved in H-bond showed in light yellow). **(B)** The overimposition of optimized geometries of YY interacting with GO (stick) and in implicit solvent (line).

Theoretical simulations (Figure 3) confirmed the preferential orientation, via hydrogen bonds formation, of the carboxylic group of each dipeptide molecule toward the hydroxyl-rich basal planes of the nanosheets (Figure 3A). On the other hand, the protonated amino groups are oriented toward the GO edges, rich in anionic carboxylic groups, by means of electrostatic forces. Moreover, the presence of additional -OH groups in YY in comparison to FF, allows for extra H-bonds that trigger the gathering of YY on the GO basal plane (Figure 3B).

**FIGURE 4:**
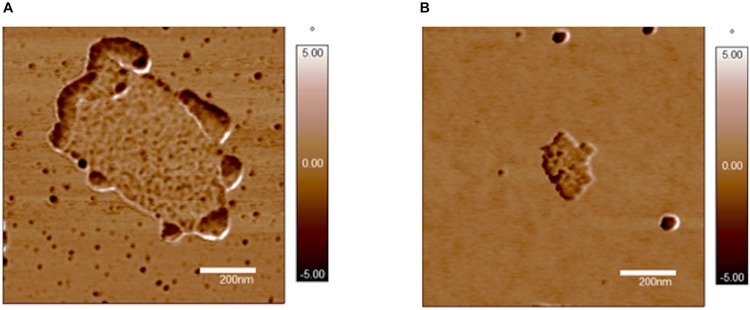
AFM phase images (xy scale = 1 × 1 μm^2^) of: **(A)** FF-GO; **(B)** YY-GO. Representative images of experiments repeated in triplicate.

Noteworthy, the phase images shown in Figure 4 display for FF + GO (Figure 4A) darker rounded-shaped flange structures protruding from the edges of a brighter GO sheet. Such regions can be assigned to hydrophobic domains of FF aggregates around the more hydrophilic polar oxygen-containing moieties on the GO (Consiglio et al., 2017). On the other hand, homogeneously distributed round features with a more uniform color with respect to the GO (and also to the hydrophilic mica substrate used for the deposition of samples) can be observed for YY + GO (Figure 4B).

To note, EPR analyses pointed to the formation of the planar structures of peptide-metal chelates that could prompt the supramolecular assembly in flat and extended domains (Supplementary Figure S5). Specifically, EPR spectra of Cu^2+^ complexes with dipeptides carried out at 150 K showed the typical pattern of metal complexes with an axial geometry (tetragonally distorted octahedral, square-base pyramidal or square-planar geometry). The values of g_| |_ (2.232 for FF and 2.234 for YY) and hyperfine coupling constant A_| |_ (184⋅10^–4^ and 186⋅10^–4^ cm^–1^ for FF and YY, respectively) resulted very similar. This finding pointed to a tetragonal geometry of the CuN_2_O_2_ chromophore for the metal complexes formed by both FF and YY. The EPR spectra of copper sulfate in the presence of GO were indicative of a CuO_4_ coordination mode. Moreover, spectra of GO showed the pattern of Mn^2+^ complex species from the exfoliation treatment with permanganate. The EPR parameters of peptide copper complexes grown in the presence of GO did not show significant changes in comparison to the peptide metal complexes, indicating a similar coordination mode of the metal ion in the dispersion of dipeptide-GO hybrids and the respective aqueous solutions.

**FIGURE 5:**
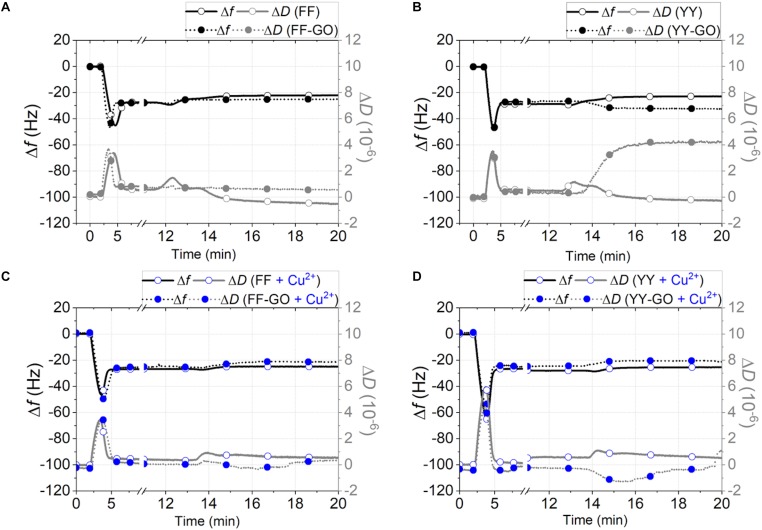
QCM-D curves of frequency (black lines, left hand side axis) and dissipation (gray lines, right hand side axis) shifts for the SLB interaction with the aggregates of: **(A)** FF (open symbols) and FF-GO (solid symbols); **(B)** YY (open symbols) and YY-GO (solid symbols); **(C)** FF + Cu^2+^ (blue open symbols) and FF-GO + Cu^2+^ (blue solid symbols); **(D)** YY + Cu^2+^ (blue open symbols) and YY-GO + Cu^2+^ (blue solid symbols). [peptide] = [Cu^2+^] = 10^–3^ M; GO = 340 μg/mL. Representative curves of experiments repeated in triplicate.

The interaction of peptide-decorated GO nanosheets with POPC SLBs, used to test the hybrid nanobiointerface with model cell membranes (Satriano et al., 2012; Yang et al., 2013; Consiglio et al., 2017; Cucci et al., 2019) was investigated by the acoustic sensing technique of quartz crystal microbalance with dissipation monitoring (QCM-D) (Figure 5). Each experimental run started in 10 mM phosphate buffer (PBS, pH = 7.4), then the addition of 100 μg/mL POPC SUVs, which firstly adsorbed intact onto the hydrophilic silica-coated QCM-D sensor, as marked by the decrease in frequency, *f*, and the increase in dissipation, *D*, respectively. Once the critical coverage was reached (roughly at *t*–5 min, corresponding to Δ*f*_*min*_ and Δ*D*_*max*_), the vesicles spontaneously brake and fused to form a homogeneous and rigid SLB (Δ*f* = ∼26 Hz and Δ*D* < 0.5⋅10^–6^) (Satriano et al., 2010). After the rinsing with PBS, the dipeptide assemblies were added (*t*∼12 min) and eventually the solution in the measurement chamber was exchanged with PBS (*t*∼22 min) for the final rinsing. The interaction of pre-formed SLBs with the nanoaggregates of FF (Figure 5A, open symbols) and YY (Figure 5B, open symbols) induced a similar response, i.e., a significant increase of frequency (Δ*f* ∼ +5 Hz) and a slight dissipation decrease (Δ*D* ∼−0.25⋅10^–6^) for both dipeptides. These findings could be representative of the insertion of peptide aggregates in the membrane, as known for Phe-Phe nanotubes at the interface with lipid vesicles (Adler-Abramovich et al., 2012; Tesauro et al., 2019).

To be noted, this interpretation is also supported by CD results discussed above, since a β-sheet configuration is adapted for insertion into the lipid membrane (Fan et al., 2018). As to the observed trend, i.e., respectively the increase of frequency and the decrease in dissipation with respect to the stabilized curves after the steps of SLB formation and rinsing, one can figure out that a stiffening of the membrane occurs, due to the intercalation of the lipophilic aromatic dipeptide molecules that perturb the packing and likely exchange with some of the lipid molecules within the bilayer. Indeed, in the approximation of a rigid adsorbed film at the surface of the sensor (which is reasonable since Δ*D ∼ 0*), and using the Sauerbrey equation (Dixon, 2008):

(1) Δm=(C/n)Δf

where C is the mass sensitivity constant (∼−17.7 ng cm^–2^⋅Hz^–1^ for a 5 MHz quartz crystal) and *n* is the overtone number, an approximate value of ∼88.5 ng/cm^2^ for apparently desorbed areal mass can be calculated. Taking into account the molecular dimensions of FF (7.2 Å × 11.6 Å × 6.0 Å) and YY (7.4 Å × 13.6 Å × 7.2 Å) in comparison with the calculated size of POPC (34 Å × 7.9 Å × 8.4 Å) (see Supplementary Figure S6), it is very likely that the intercalation of dipeptide aggregates prompt the removal of some lipid molecules. In the case of free FF and YY dipeptide aggregates, the most probable process occurring at the interface is that the peptide inserts at the outer leaflet in the interface between the headgroup and tail core, thus resulting in a slight decrease in the lipid packing order of the bilayer (Δ*f* increase), and, in turn, a membrane stiffening (Δ*D* decrease) (Nielsen et al., 2019).

As to FF-GO, a minor decrease in *D* accompanied by a negligible decrease in *f* (Figure 5A, solid symbols). This result implied that the bilayer surface rearranged without significant poration and wrinkling due to peptide insertion. Very differently, upon YY-GO addition (Figure 5B, solid symbols), the membrane surface likely became porous and uneven, as pointed out by the continue increase of dissipation (up to Δ*D ∼* 4⋅10^–6^), indicative of a dramatic morphological transition (Fan et al., 2018). According to AFM, these results could be explained in terms of the preferential orientation of GO nanosheets with basal planes parallel to the top leaflet of the membrane. Hence, the interaction between the dipeptide molecules and the lipid membrane was minimized for Phe-Phe (preferentially grown along the GO edges) and instead enhanced for Tyr-Tyr (primarily assembled at the basal plane), respectively. Therefore, the different molecule gathering effect at the GO nanosheets (i.e., FF or YY preferentially accumulated at the edges or the basal planes of GO, respectively) can be reflected in different perturbation on the ordering of the POPC SLB (Griffith et al., 2014), with the YY-GO/SLB or FF-GO/SLB interfaces most likely being represented by a “carpet-like” or “barrel-like” models (Kumar et al., 2018).

To note, for the addition of FF + Cu^2+^ (Figure 5C, open symbols) or YY + Cu^2+^ (Figure 5C, open symbols) samples, where comparable flat and extended domains were found from AFM imaging, not as much of SLB perturbation was observed (Δ*f ∼* + 2 Hz and Δ*D ∼* 0 for both cases). As to FF-GO + Cu^2+^ (Figure 5C, solid symbols) and YY-GO + Cu^2+^ (Figure 5D, solid symbols), the increase in frequency that plateau for Δ*f ∼*+3 Hz and Δ*f ∼*+4 Hz, respectively for FF and YY, is accompanied by a decrease in *D* that, roughly 3 min after the addition, recover to similar values to those of the stabilized curves before the addition. These findings confirm a critical role of the metal in the peptide interaction with the lipid bilayer, including the decreasing of deintercalation effect (Das et al., 2017) and increasing of order within the lipid bilayer (Pabst et al., 2007).

**FIGURE 6:**
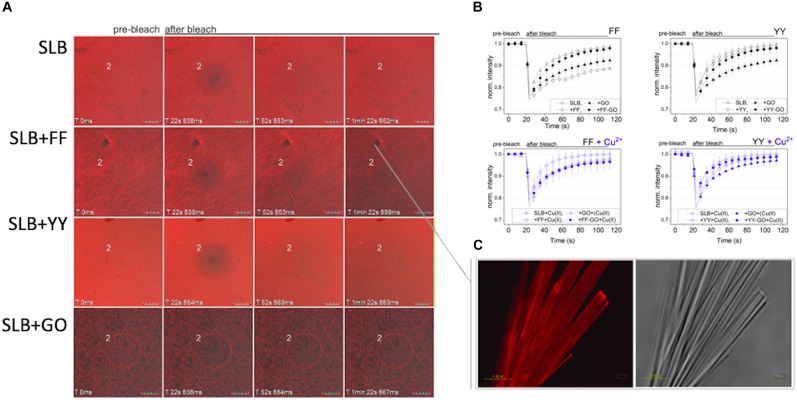
Representative confocal micrographs (**(A)**, scale bar = 10 μm) and intensity curves **(B)** for the FRAP experiment of rhodamine-labeled (in red) SLBs, both untreated and after 15 min of exposure to the peptide-GO samples assembled in the absence (black) or the presence (blue) of CuSO_4_ (triangle = bare SLB; circles = treated SLBs; open symbols = free dipeptides; solid symbols = GO-driven assemblies). **(C)** A confocal (left hand side) and optical bright field (right hand side) micrograph of FF nanotubes after the interaction with rhodamine-labeled lipids; scale bar = 4 μm.

Fluorescence recovery after photobleaching (FRAP) experiments confirmed the different interaction between the dipeptide nanoaggregates and SLB, as well as a modulating effect by the presence of GO and/or Cu^2+^, as measured in terms of the lateral diffusion coefficients of the lipids within the membrane (Figure 6). To note, the SLB fluidity (with a calculated lipid lateral diffusion coefficient, of ∼34 μm^2^⋅s^–1^, see Supplementary Material) significantly decreased after the membrane exposure to FF (∼24 μm^2^⋅s^–1^) and GO (∼20 μm^2^⋅s^–1^), but exhibited modulated levels of membrane rigidity after the treatment with the different peptide-GO samples. In particular, the poration of the SLB due to FF peptide insertion was clearly visible (Figures 6A,C), whereas an enhanced recovery after photobleaching was especially exhibited in the presence of Cu^2+^ that favored the lipid lateral diffusion within the membrane.

Proof-of-work experiments on the different interaction of the peptide aggregates grown in the diverse experimental conditions with true cell membranes were performed. In particular, a dose-response experiment of MTT assay was carried out at a short time of incubation (*t* = 1 h) with two cancer cell models, namely human neuroblastoma (SH-SY5Y line) and prostate (PC-3 line), which are known to exhibit different cytotoxic response by drug incorporation into the cell membrane (D’Eliseo and Velotti, 2016). The rationale of this experiment was to scrutinize the early effects on the peptide-cell membrane interaction as in some protocol studies for small cell penetrating peptides, which are able to strongly interact with the cell membrane inducing a significant reduction of the cell viability even after 1h of incubation (Bocsik et al., 2019).

**FIGURE 7:**
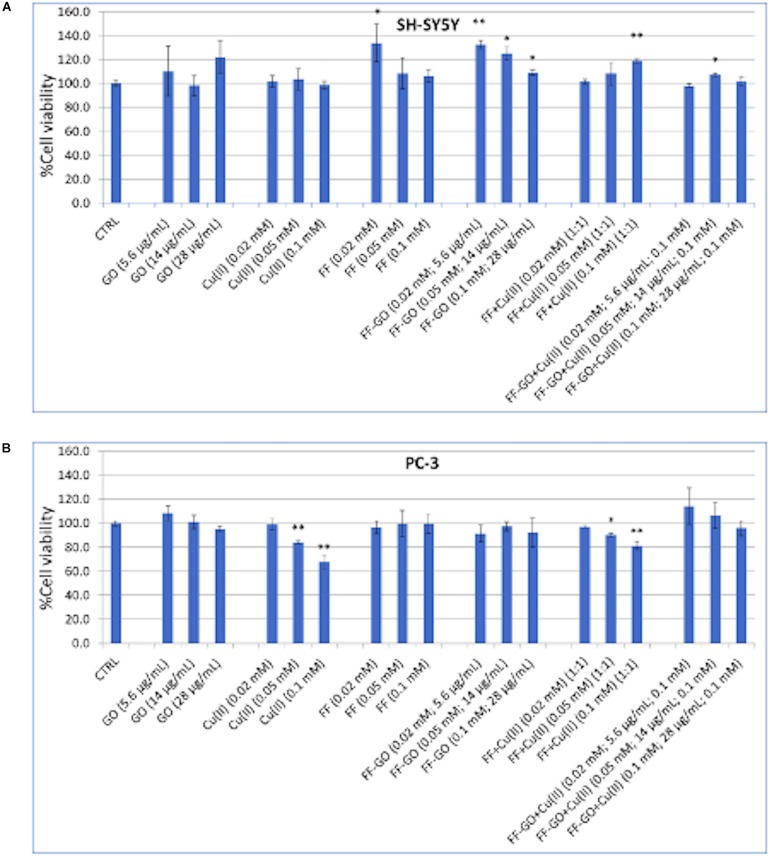
Cell viability of SH-SY5Y **(A)** and PC3 **(B)** cells after 1 hr treatment with for the dipeptide (1⋅10^–4^, 5⋅10^–5^, and 2⋅10^–5^ M), Cu^2+^ (1⋅10^–4^, 5⋅10^–5^, and 2⋅10^–5^ M), GO (28, 14, and 5.6 μg/mL) or the hybrid FF-GO, FF + Cu^2+^and FF-GO + Cu^2+^ samples. (* = *p* < 0.05, ** = *p* < 0.01, vs. CTRL, One-way ANOVA).

MTT results (Figure 7) on SH-SY5Y and PC-3 cells show that the cellular treatments with positive controls of GO or Cu^2+^ resulted in different effects on the two different cell lines. Specifically, GO did not affect significantly the cell viability of both cancer cell lines at the tested conditions. On the other hand, the cell treatment with copper ions did not induce cytotoxicity on neuroblastoma cells, while a significant reduction of cell viability is observed at the higher concentrations of 0.1 and 0.05 mM for PC-3. Graphene and its derivatives have a potential cytotoxic effect depending on their size and concentration (Jarosz et al., 2016). For instance, GO can induce upon the interaction with the cell membrane, changes in the cell morphology, destruction of membrane integrity and DNA damage due to the increased production of reactive oxygen species (Chatterjee et al., 2014). Moreover, copper ions were found to increase the viability SH-SY5Y in a dose-response mode (Travaglia et al., 2011). Contrarily, copper-induced cytotoxic effects due to reactive oxygen species formation were observed after short time treatment in PC3 cell lines (Safi et al., 2014).

As to the cellular treatments with FF, no cytotoxic effects were observed on both the cell lines instead, instead a significant increase of viability was detected on SH-SY5Y at the lowest concentration tested for FF and, in a dose-dependent manner, for the hybrid FF-GO. No significant difference with respect to the untreated cells were observed for PC-3 treated either with FF or FF-GO samples. Finally, as to the cellular treatments with FF + Cu^2+^ or FF-GO + Cu^2+^, roughly ∼ 20% of increased viability was exhibited by neuroblastoma cells (after the incubation with the FF + Cu^2+^ at the highest tested peptide concentration). On the other hand, a dose dependent cytotoxicity was still observed in PC-3 cells, with a similar but less pronounced trend as the treatment with Cu^2+^ alone. In summary, these results point to a cell-dependent response, with different trends of increase or decrease in cell viability by the various cellular treatments with dipeptide-GO, dipeptide+ Cu^2+^ or dipeptide-GO+ Cu^2+^ systems.

## Conclusion

In this work we addressed the decoration of graphene oxide nanosheets by aromatic dipeptide nanostructures, self-assembled both in the absence and in the presence of copper ions. The spectroscopic and microscopic characterisation (CD, UV–visible, fluorescence, EPR, AFM) pointed to: (i) a strong interaction between the GO and the dipeptides, as monitored by the bands of π → π^∗^ transition, characteristic of peptide aggregates by the molecular stacking in beta-sheet conformation; (ii) a triggering effect by the copper ions on the peptide self-assembly process, by the formation of metal complexes, as well as on the preferential gathering of the peptide aggregates onto the edges and/or the basal planes of the GO nanosheets. Theoretical calculations confirmed the possibility to tune, by means of electrostatic vs. hydrophobic interaction forces, the growth of the peptide aggregates at the interface with GO.

QCM-D and FRAP experiments with SLBs and cellular experiments on two model cancer cells, pointed out the tuneability of the interaction between the peptide-decorated GO nanosheets and cellular membrane, as demonstrated in terms of viscoelastic properties and lateral diffusion of the lipids within the membrane as well as by proof-of-work *in vitro* assays of cell viability after short time of treatment with the various systems. Hence, the surface tailoring of GO by self-assembled aromatic dipeptides (FF and YY) offered a simple route for a tuned interaction between these 2D nanomaterials and lipid membranes, thus remarking their potentialities as versatile nanoplatforms at the hybrid biointerface for nanomedicine and theranostic applications.

## Data Availability Statement

All datasets generated for this study are included in the article/Supplementary Material.

## Author Contributions

GTr and VC carried out the experiments and drafted the manuscript. LC, FA, and GTa carried out the experiments (CD, ThT, and EPR, respectively). GF helped with data analyses and modeling. DL and CS conceived the original idea, supervised the project, revised and wrote the final manuscript.

## Conflict of Interest

The authors declare that the research was conducted in the absence of any commercial or financial relationships that could be construed as a potential conflict of interest.
