# Supplementary material for: Graphene Oxide Nanosheets Tailored With Aromatic Dipeptide Nanoassemblies for a Tuneable Interaction With Cell Membranes
Source: Front Bioeng Biotechnol. 2020 May 8;8:427. doi: 10.3389/fbioe.2020.00427 (PMC7227426; doi:10.3389/fbioe.2020.00427)
Supplement: Supplementary file 1 [file Presentation_1.pdf]

Supplementary Material

Graphene oxide nanosheets tailored with aromatic dipeptide nanoassemblies for a tunable interaction with cell membranes

Giuseppe Trapani [1†§], Viviana Carmela Linda Caruso[2†], Lorena Maria Cucci[2], Francesco Attanasio[3], Giovanni Tabbì[3], Giuseppe Forte[4], Diego La Mendola[5]* and Cristina Satriano[2]*

[1] Scuola Superiore di Catania, University of Catania, 95123 Catania, Italy.

[2] Nano Hybrid BioInterfaces Lab (NHBIL), Department of Chemical Sciences, University of Catania, 95125 Catania, Italy.

[3] Institute of Crystallography − National Council of Research, 95123 Catania, Italy.

[4] Department of Pharmaceutical Sciences, University of Catania, 95125 Catania, Italy.

[5] Department of Pharmacy, University of Pisa, 56126, Pisa, Italy.

*** Correspondence:**
Prof. D. La Mendola, lamendola@farm.unipi.it; Prof. C. Satriano, csatriano@unict.it

[§] Current affiliation: Bioactive Materials Laboratory, Max Planck Institute for Molecular Biomedicine, 48149 Münster, Germany.

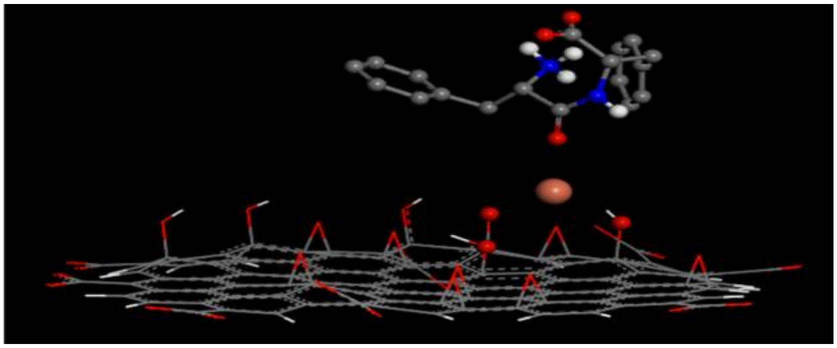

Supplementary Figure 1. A schematic representation of Phe-Phe dipeptide at the interface with a GO nanosheet in the presence of Cu(II).

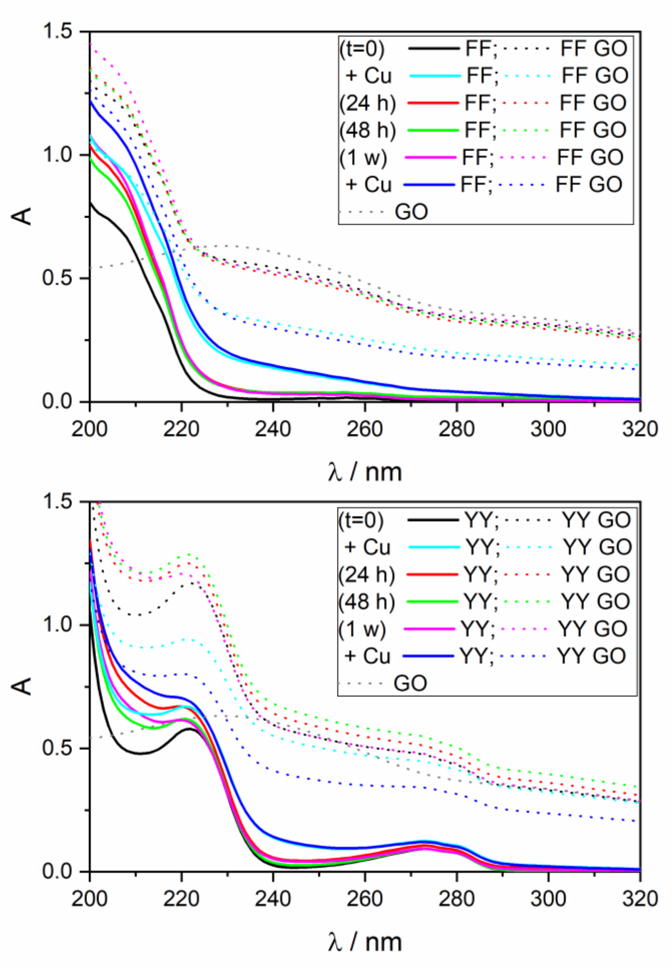

Supplementary Figure 2. UV-visible spectra of free dipeptide (solid curves, $5x10^{-5}$ M solution in water) and GO/dipeptide (dotted curves, GO concentration = 17 μg/mL) assemblies at different aging times since dissolution. For t=0 and t=1 week the spectra of the samples added with $CuSO_4$ ($5x10^{-5}$ M) are included for comparison.

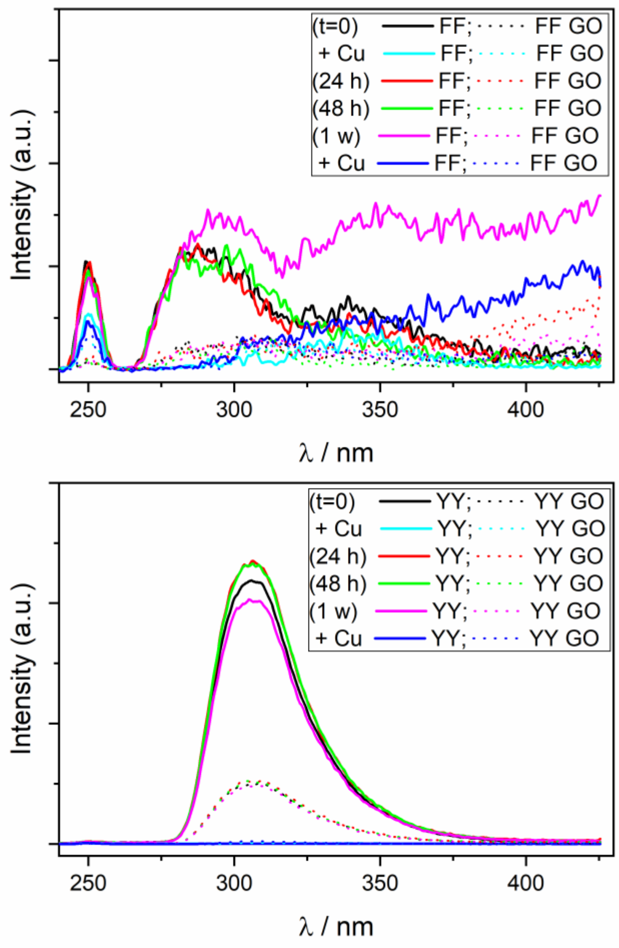

Supplementary Figure 3. Emission spectra (excitation wavelength = 230 nm) of free dipeptide (solid curves, 5x10-5 M solution in water) and GO/dipeptide (dotted curves, GO concentration = 17 μg/mL) assemblies at different aging times since dissolution. For t=0 and t=1 week the spectra of the samples added with $CuSO_4$ (5x10-5 M) are included.

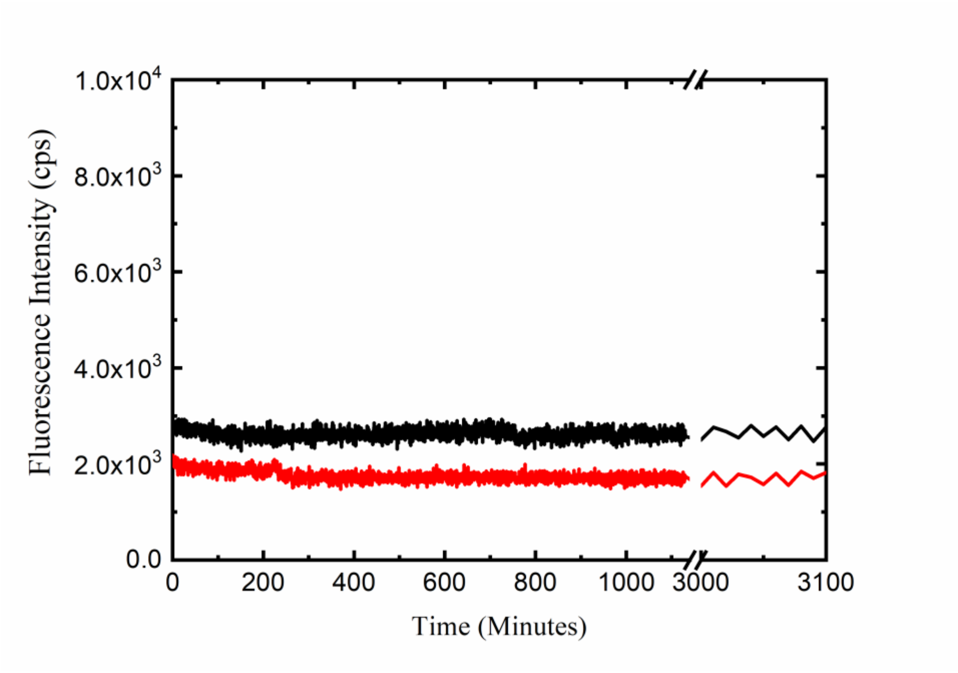

Supplementary Figure 4. Time dependent Th-T fluorescence profile for FF-GO (50 μM) in 10 mM phosphate buffer pH = 7.4 (red line) and H_2O (black line) recorded at 25°C for 2 days.

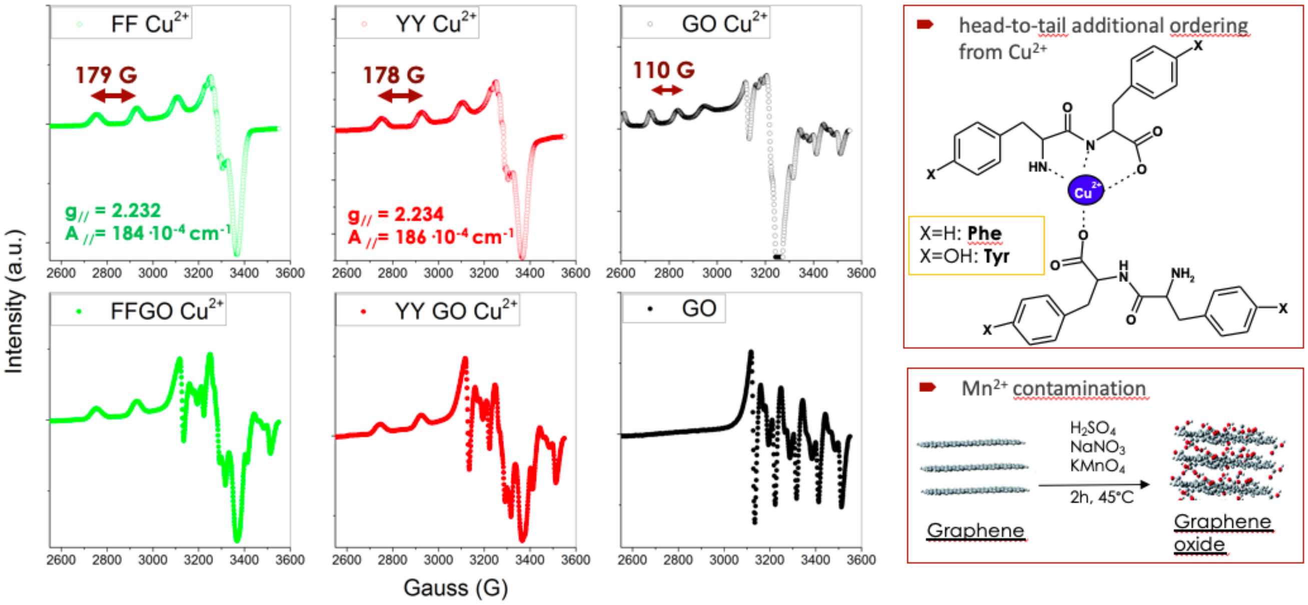

Supplementary Figure 5. EPR spectra of FF-Cu^{2+}, YY-Cu^{2+} and the corresponding GO-based platforms. Experimental conditions to record the spectra were as follows: 150 K in MQ-H_2O; $[dipeptide]=10^{-3}M$, GO concentration = 340 $\mu g/mL$, $[Cu^{2+}]:[dipeptide]$ = 1:1. The panels in the right hand side show one of the possible coordination environments of the copper chelates in the

peptide-copper complexes as well as the interlayer ions of manganese ‘contamination’ detected in the EPR spectra.

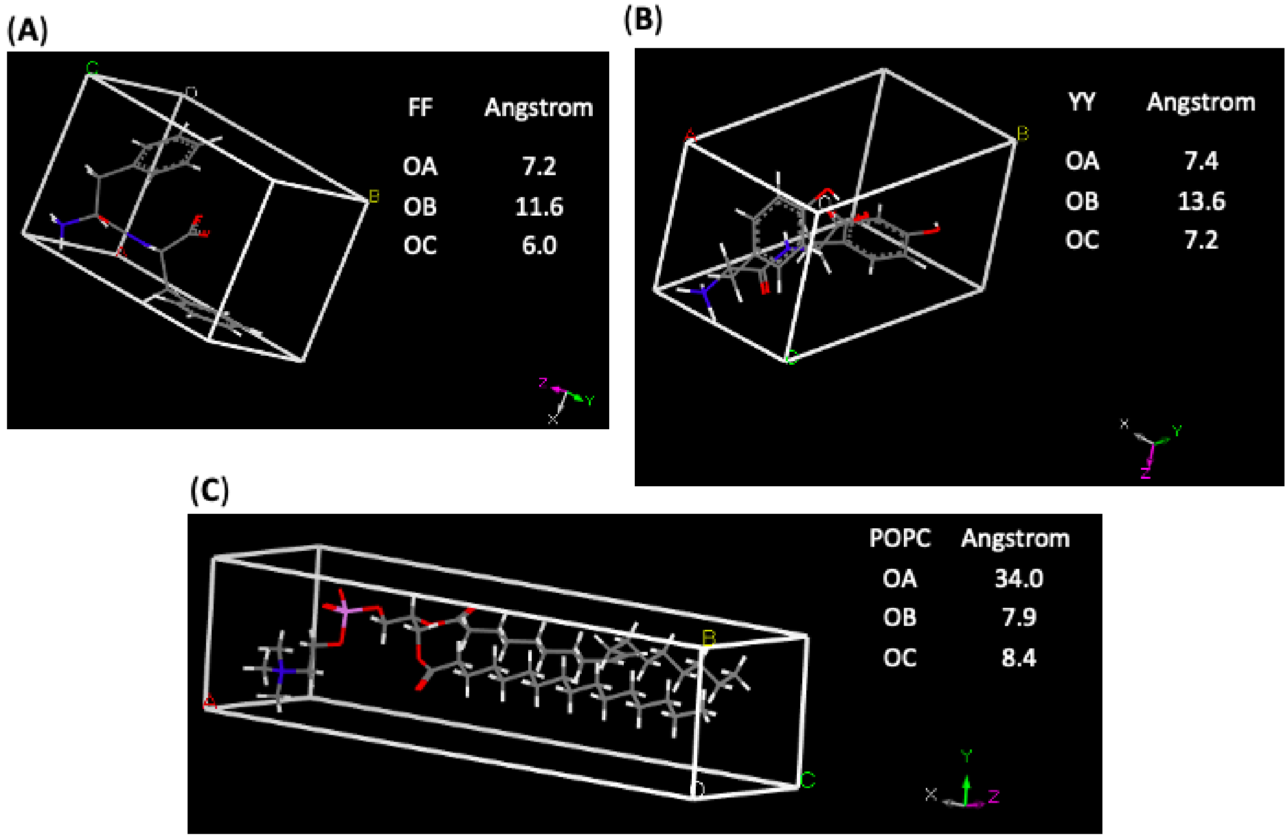

Supplementary Figure 6. Optimized structures of: (**A**) FF, (**B**) YY, (**C**) 1-palmitoyl-2-oleoyl-sn-glycero-3-phosphocholine (POPC).
